# Correlation Between Pulmonary Sclerosing Pneumocytoma Features and MSCT Imaging Manifestations in 34 Patients: Implications for Precision Medicine

**DOI:** 10.3389/fmed.2021.650996

**Published:** 2021-03-18

**Authors:** Gen Xu, Zhaoyu Wang, Zeng Xiong, Manqiu Li, Weijun Luo, Yong Xu, Tang Min

**Affiliations:** ^1^Department of Radiology, First People's Hospital of Pingjiang County, Yueyang, China; ^2^Shanghai Hengdao Medical Pathology Diagnostic Center, Shanghai, China; ^3^Department of Radiology, Xiangya Hospital, Central South University, Changsha, China; ^4^Department of Pathology, First People's Hospital of Pingjiang County, Yueyang, China

**Keywords:** pulmonary sclerosing pneumocytoma, pathology, multi-slice computed tomography, image pathological control, diagnosis

## Abstract

**Objective:** To identify and analyze the multi-slice computed tomography (MSCT) imaging manifestations and clinicopathological features of PSP to improve the preoperative and intraoperative diagnosis of the disease.

**Method:** This was a retrospective study conducted on the imaging and clinicopathological data of the PSP patients treated in two major hospitals in China from October 2001 to December 2019. The locations of lung lesions, clinical symptoms, surgical complications, MSCT imaging features, and the corresponding relationship with clinicopathological features were assessed. Then, a new diagnostic approach was defined and used to train imaging and pathological doctors (experimental group). Then, the diagnostic accuracy of the experimental group was evaluated in preoperative and intraoperative diagnosis of PSP.

**Results:** Thirty-four PSP cases were analyzed (mean: 51.42; range: 39–69 years old). The peripheral type was more common, while 92% of the lesions located in the middle lobe of the right lung and the lower lobe of bilateral lungs. The shortest lesion edge-pleura distance ranged 0 to 30 mm and 46% of the lesions (16/34) were attached to the pleura, 62% (21/34) located at 0–5 mm, 92% (31/34) within 20 mm from the pleura. Diameters of the lesions ranged 8.58 to 68.41 mm, while most of them were 20-40 mm. All lesions showed enhancement, and 97% (33/34) were unevenly enhanced. PSP volume was negatively correlated with the total degree of enhancement (*r* = −0.587, *p* < 0.01), and the volume difference between the obvious enhancement zone and the slight enhancement zone (*r* = −0.795, *p* < 0.01). Welt vessel sign was observed in 61.7% (21/34) of cases, and none of welt vessels entered into the lesions. Vascular-like enhancement area inside the lesion showed no significant correlation with the welt vessels outside the lesion, and no case showed entrance of bronchus into the lesion. The trained experimental group showed significantly greater diagnostic accuracy than the control group. In particular, the accuracy rate of intraoperative frozen section diagnosis was 60% higher in the experimental group than the control group.

**Conclusion:** PSP has characteristic imaging manifestations, which can be utilized to improve the preoperative and intraoperative diagnostic coincidence rate of PSP.

## Introduction

Pulmonary sclerosing pneumocytoma (PSP), formerly known as pulmonary sclerosing hemangioma (PSH), was first reported by Liehow and Huhell in 1956 as an uncommon lesion with an uncertain origin (1). PSP manifests a pulmonary neoplasm with a complicated and an undefined histogenesis. It was later found to be a tumor in the lung parenchyma that originated from type II alveolar epithelial cells and had a successful clinical process (2, 3). World Health Organization (WHO) (2015) has histologically classified lung tumors and renamed it as pulmonary sclerosing pneumocytoma and identified as a lung adenoma (4). PSP has been reported as the most common benign tumor in the lung, while the incidence rate was relatively high in East Asia. More than 80% of cases occurred in middle-aged women > 50 years of age (5, 6). Since most PSPs were discovered by accident, there were no typical reliable clinical manifestations, and the accuracy rate of the intraoperative frozen section (FSS) was 44.1%, while the delay rate was 15.3% (4). Despite of benign nature, PSP represents a diagnostic challenge due to its controversial etiology and biologic behavior, as well as the diversity of pathohistological findings. The main diagnostic challenge of PSP is that it represents a diversity of pathohistological findings (7). Therefore, the diagnostic accuracy of the preoperative imaging is particularly important. Moreover, definitive diagnosis of PSP currently relies mainly on surgical pathology because the preoperative imaging modalities show poor specificity.

Computed tomography (CT) imaging modalities have shown significant diagnostic and differential diagnostic values for different lung disorders (8–12). PSPs comprise of 4 major histologic patterns with different proportions including hemangiomatous, papillary, sclerotic, and solid. These patterns manifest demonstrate various CT features depending on the composition of PSP (13–18). Studies have demonstrated that CT imaging particularly multi-slice CT (MSCT) imaging offer promising diagnostic values for early or differential diagnosis of different pulmonary lesions and disorders including tuberculosis and diffuse peritoneal lesions (12, 16, 18–20). Meng et al. retrospectively analyzed CT imaging manifestations of three common diffuse peritoneal lesions to evaluate the diagnostic and differential diagnostic value of CT in diffuse peritoneal lesions (19). They analyzed features of CT imaging in patients with diffuse peritoneal lesions (72), including 16 cases with tuberculous peritonitis (*n* = 16), peritoneal metastasis (21), and peritoneal mesothelioma (22). They reported that specific features of CT images have significant diagnostic values for the differential diagnosis of peritoneal diffused lesions. These features are the location of peritoneal lesions, morphology, ascitic fluid and lymph nodes. He et al. retrospectively analyzed the CT features of PSP in 33 patients (confirmed by pathology) to improve the correct rate of CT in diagnosis of PSP. They scanned all patients with plain CT scan and in 29 patients they performed simultaneous CT enhanced scan in addition (18). They reported that PSP is often manifested as a single round soft tissue density nodule. Enhanced scan is characterized by obvious enhancement and continuous enhancement of delayed scan, with some characteristic concomitant signs. They concluded that by considering the variations in CT images associated by age and sex, the diagnostic accuracy of CT images could be improved (18). Reviewing the findings of CT imaging studies in PSP cases it can be clarified that PSP usually manifests as a well-defined, juxta-pleural nodule with strong and homogeneous enhancement on CT (13–16, 23).

Few studies have so far evaluated CT findings of PSP and analyzed the imaging features to determine its pathologic correlation (14, 22, 23). However, the main issue with the previous studies is that the sample size was very small and more importantly the clinical practice still suffers limitations in diagnosis of PSD. These limitations are mainly because the currently used preoperative imaging modalities based on these studies suffer poor specificity and slow process.

The present study was aimed to summarize and evaluate the corresponding relationships between the clinical-pathological features and the MSCT imaging manifestations of PSP in order to improve the preoperative and intraoperative diagnosis of the disease.

## Materials and Methods

### Study Design and Population

All experimental procedures of this study were approved by the local ethics committee of Xiangya Hospital, Central South University, Changsha, China (Code: 202006082) that are in complete accordance with the ethical standards and regulations of human studies of the Helsinki declaration (2014). A total of 34 cases of PSP, who were diagnosed with surgical resection, pathology, and immunohistochemistry, were selected from Xiangya Hospital of Central South University and the First People's Hospital of Pingjiang County, Hunan Province, China from October 2001 to December 2019. The MSCT and pathological data of patients were reviewed. The related information, including gender, age, tumor size, tumor location, pathological features, and imaging manifestations, were recorded. None of the selected cases were combined with malignant changes, and the cases of intra-lung metastasis of lung cancer or metastatic lung cancer were excluded. The basis and classification of the pathological diagnosis were referred to WHO (2015) for the histological classification of lung tumors.

### Preparation of Tissue Samples

**Figure 1:**
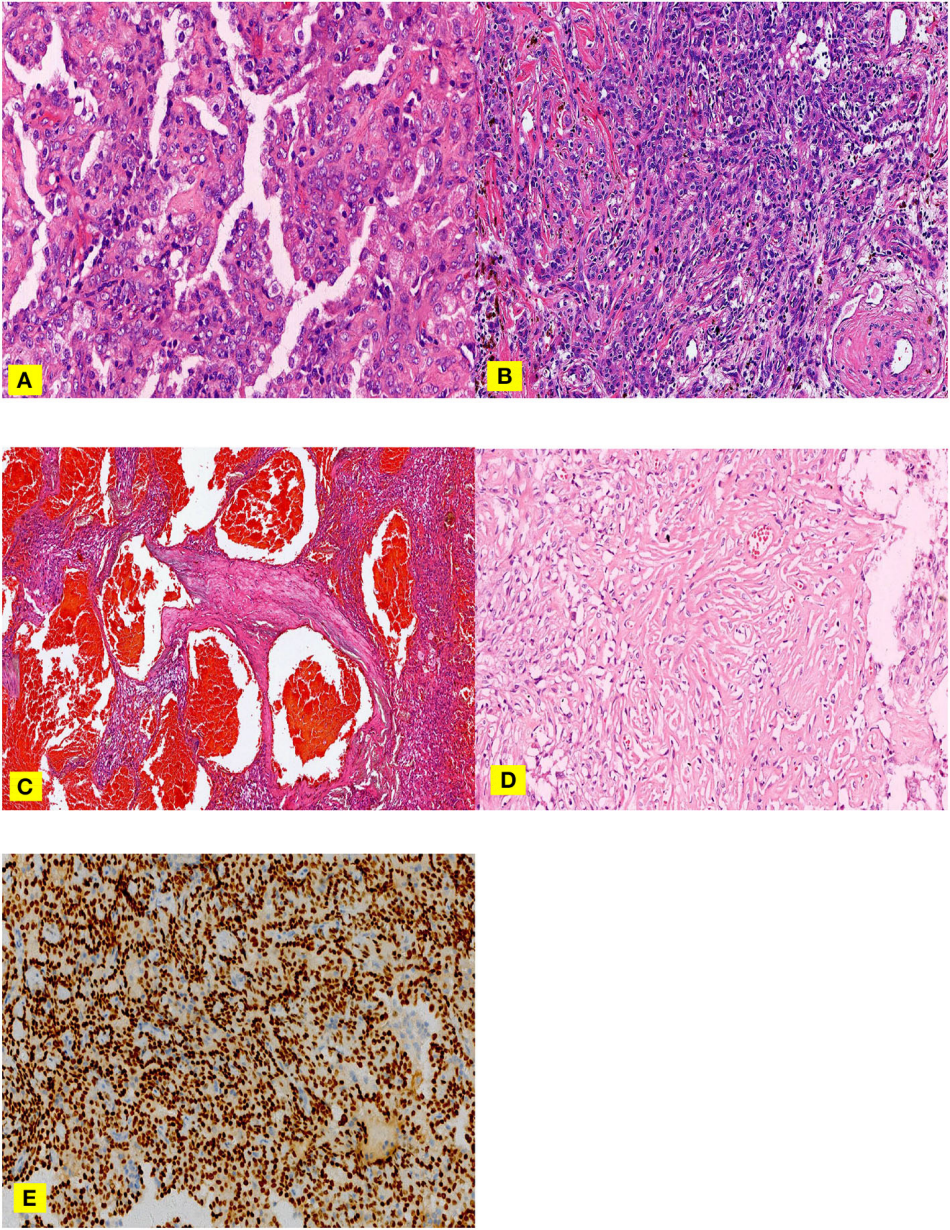
**(A–E)**. H &E staining **(A–C)**, nipple area: surface cubic cells covered on the nipple surface and polygonal cells in the nipple interstitium can be observed **(A)** Solid area: cells were dense, the size was the same, and it was in the shape of a sheet **(B)** Hemangioma-like area: the vasculature-like space dilation, which filled with a large amount of fresh red blood cells. **(C)** Sclerosing area: a large amount of collagen fibers was observed, in which various inflammatory cells and adenoid-like structures lining with a small amount of surface cubic cells. TTF-1 **(E)** Surface cubic cells and polygonal cells were positive.

All specimens were fixed with 3.7% neutral formaldehyde, dehydrated routinely, and embedded in paraffin to make wax blocks. A paraffin block with tumor tissues was selected, and each paraffin block was cut into a 4 μm thick section for Hematoxylin and eosin (H&E) staining procedure (Figure 1). The slice was then observed under a light microscope. They were read by two attending physicians with relevant experience in the pathology department. If any inconsistencies found, then a third deputy chief physician in the pathology department was jointly discussed and decided.

### MSCT Scanning Technology and Parameters

For the MSCT imaging we used the most recommended imaging protocol for focal and diffuse lung diseases recommended in literature and according to the instructions of the manufacturer (24, 25). pulmonary lung The GE LightSpeed 64-slice Volume Computed Tomography (VCT) (GE Healthcare) with Nemoto high-pressure injector were used for image acquisition with the scan parameters of 120 kV and 10–400 mA. Automatic tube current modulation (ATCM) technology was used in which the noise index (NI) value was 15, the screw pitch 0.984: 1, speed 0.5 s, collimation width 64 × 0.625 mm, scanning layer thickness/layer interval 5 mm, reconstruction layer thickness 1.25 mm, and reconstruction interval was 0.5 mm. The subject was taken a supine position with feet entered first, the hands were straight up and raised with support. The scanning range was from the entrance of the rib cage to the bottom of the lungs. The scan was completed under a state of deep breath inhalation. Non-ionic contrast agent ioversol (concentration was 320 mg I/ml) was used, 60–75 ml contrast agent was injected through the right median cubital vein using a 20 G indwelling needle at an injection rate of 3.0 ml/s, followed by an injection of 30 ml normal saline at the same flow rate. Two-phase scanning was performed, while the arterial phase delay time was 25 s, and the parenchyma phase delay time was 60–70 s. After the scanning was completed, the reconstructed cross-section thin slice images were transferred to the AW4.5 workstation for post-processing, including multi-planar reconstruction (MPR), planar reconstruction (CPR) and volume rendering (VR). In the imaging protocol, the window settings (window-width and window-level values) were defined as follows. For mediastinal imaging (window width: 350 Hounsfield unit (HU); window-level: 40 HU) and for lung imaging (window width: 1200 HU; window level: −600 HU).

### Evaluation and Measurement of MSCT Imaging Manifestations

#### Evaluation Methods

The MSCT image data of 34 patients were reviewed by two attending physicians with specific expertise in the radiology department. If any discrepancies found, then a third Deputy Chief Physician in the radiology department was jointly discussed and decided.

Similarly, the pathological section data of 34 patients were reviewed by two attending doctors in the pathology department with relevant experience and followed the same protocol as above.

#### Contents and Standards of Image Evaluation

The collected MSCT images were evaluated as per the following procedures.

1. The tumors were examined by their site and location, the shortest distance between the edge of the lesion and the pleura, morphology, size, density, etc.
2. External signs of tumors on MSCT have been identified by welt vessel sign, halo sign, air crescent sign, calcification, necrosis, cystic changes, lobulation sign, atelectasis, pleural traction and thickening, enlargement of hilar and mediastinal lymph nodes, etc.
3. Internal features of tumors on MSCT were evaluated using the image analyses. According to the net increased CT value, that was, the absolute value of the difference between the CT values of enhanced scan and the plan scan, referring to the Swensen standard (26), it was divided into a non-enhanced area (increased CT value < 15 Hu), a marginally enhanced area (CT value increased by 15–25 Hu), a moderately enhanced area (CT value increased by 25–45 Hu), and a significantly enhanced area (CT value increased by> 45 Hu).

#### Contents and Standards of Pathological Evaluation

The pathological evaluations of the samples were conducted using conducted as follows:

1. Basic information on the size, location, central or peripheral type, and overall morphology of the tumors were examined and recorded by an expert oncologist.
2. Microscopic histopathological observations were done on H&E stained transverse section of the samples included two types of cells (surface cubic cells and polygonal cells) and four structures (nipple area, sclerosing area, solid area, and hemangioma-like area) while atypia cell was not visible, but foam cells were often present.
3. Immunohistochemical characteristics were achieved by diffuse TTF-1 positive expression when CK7 was expressed only on the surface of the epithelium.

### Measurements

The measurements were performed as per the following steps:

1. When the long diameter of the lesion was higher than 10 mm, three consecutive levels were measured. If the CT value was >10 HU, the median value was considered. If the CT value of the three levels was equal or < 10 Hu, then, the average value was considered.
2. If the long diameter of the lesion was < 10 mm, the CT values of only 1–2 layers were measured, and the average value was taken.
3. When the range-of-interest (ROI) was >70% of the lesion, the selected measurement area avoided the necrosis, cystic, and calcification regions as much as possible.

**Figure 2:**
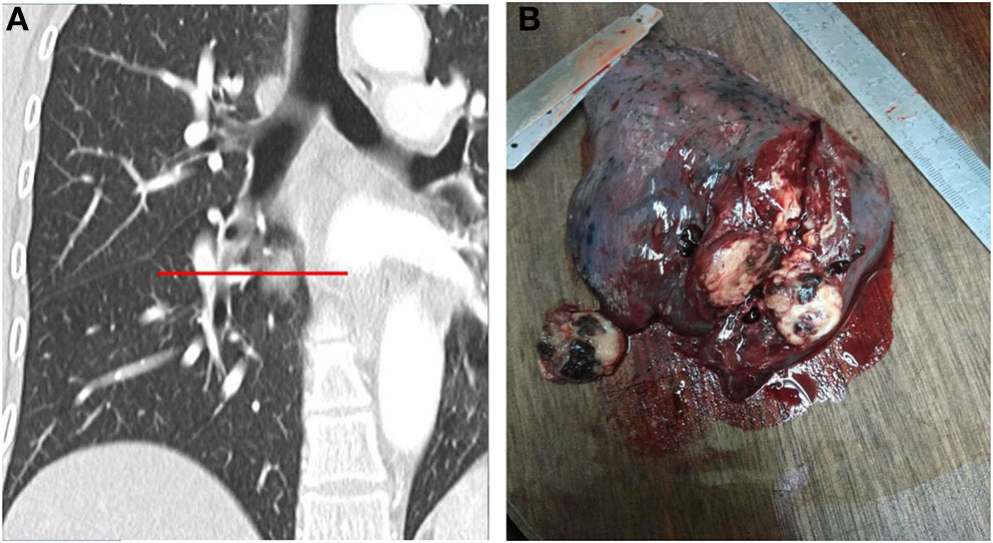
**(A,B)**. MSCT multiplanar reformation and simulation of gross specimen cut **(A)**. The gross specimen of PSP had a clear boundary and was easy to disassociate from the surrounding lung tissues. The cut surface was apricot-white-gray to yellow-brown, with a medium texture, and dark red bleeding areas can be observed **(B)**.

Considering the above terms and criteria, the collected MSCT images were evaluated and the imaging features were recorded and sorted in the specific tables for further analyses (Figure 2). For each variable, three independent measurements were performed, and the averaged value was considered along with the standard deviation (SD).

#### Performance of a New Diagnostic Method in Clinical Setting

The performance and accuracy of the new diagnostic method were evaluated as per the following scenario:

5.1. Five radiologists were selected as an experimental group (one Deputy Chief Physician, two attending physicians, and two physicians), and the new diagnostic approach summarized above was used for teaching. Another five radiologists were selected as the control group (1 Deputy Chief Physician, two attending physicians, and two physicians), and the traditional diagnostic method was used for teaching. Both groups of physicians had a double-blind model, qualifications, years of experience, degrees, and proportions were the same. There was no statistically significant difference in composition ratios of age and gender. After the lecture, the two groups of doctors read the same set of PSP (pathologically confirmed) image data respectively and diagnosed the images. The coincidence rate of image-pathological diagnosis has been statistically analyzed. There were 10 cases in each group, 1 point was awarded for each correct diagnosis.
5.2. Three radiologists were selected as an experimental group (1 Deputy Chief Physician and two attending physicians), and the new diagnostic method, summarized earlier was taught to them. The main features of the images, important to reach a diagnosis along with the samples were used to train the experimental group as the new diagnostic imaging. Another three radiologists were selected as the control group (1 Deputy Chief Physician and two attending physicians), and the standard diagnostic approach was used for teaching. Both groups of doctors used a double-blind model, qualifications, years of practice, degrees, and proportions were the same, and there was no statistically significant difference in composition ratios of age and gender. After the training course, the two groups of doctors read the same set of PSP (pathologically confirmed) image data respectively and then diagnosed the images. The coincidence rate of image-pathological diagnosis has been statistically analyzed. There were 10 cases in each group, 1 point was awarded for each correct diagnosis.

## Statistical Analysis

6.1. Chi-square test and Fisher's exact probability method were used to evaluate the relationship between the type and extent of the MSCT enhancement, pathological characteristics, and various pathological tissue structures of the PSP. A multivariate regression model (Logistic model) was used to analyze the correlation between clinicopathological features and MSCT imaging of PSP and age, gender, location, the shortest distance between lesion margin and pleura, and histological types. All tests were two-sided, while *P* < 0.05 was considered as statistically significant. Statistical package for social sciences (SPSS) (version 21) package was used for statistical analysis.

## Results

### Clinical Characteristics

A total of 34 patients with PSP were selected. There were 1 male (3%) and 33 females (97%), with a minimum age of 39 years, maximum age of 69 years, the average age of 51.42 years, and a median age of 52 years. The age was normally distributed. Among them, 14 cases (41.17%) were < 50 years of age, and 20 cases (58.83%) were more than or equal to 50 years of age. Except for two cases of long-term cough and one evidence of blood in sputum, the remaining 31 cases were all identified by physical examinations. The median age of this group of cases was older than that reported in some literature at home and abroad, which may be because the recommended age of physical examination for LDCT was > 40 years old in Hunan Province, China. Since most PSPs were discovered by chance and did not have traditional and reliable clinical manifestations, the present study did not consider the impact of clinical signs and symptoms of PSP on the results of the research.

### MSCT Plain Scanning Manifestations

#### Distribution

The results of the examination showed that all the 34 cases were single isolated lesions. They were located in the middle lobe of the right lung in 10 cases, the inferior lobe of the right lung in 10 cases, superior lobe of the left lung in 2 cases, inferior lobe of the left lung in 11 cases, and hilus pulmonis in 1 case.

#### Size

The long diameter of the lesion ranged from 8.58 to 68.41 mm, the small one was 8.58 mm, and the largest was about 68.41 mm. Most of the lesions ranged from 20 to 40 mm.

#### Lesion Margin-Pleura Shortest Distance

**Figure 3:**
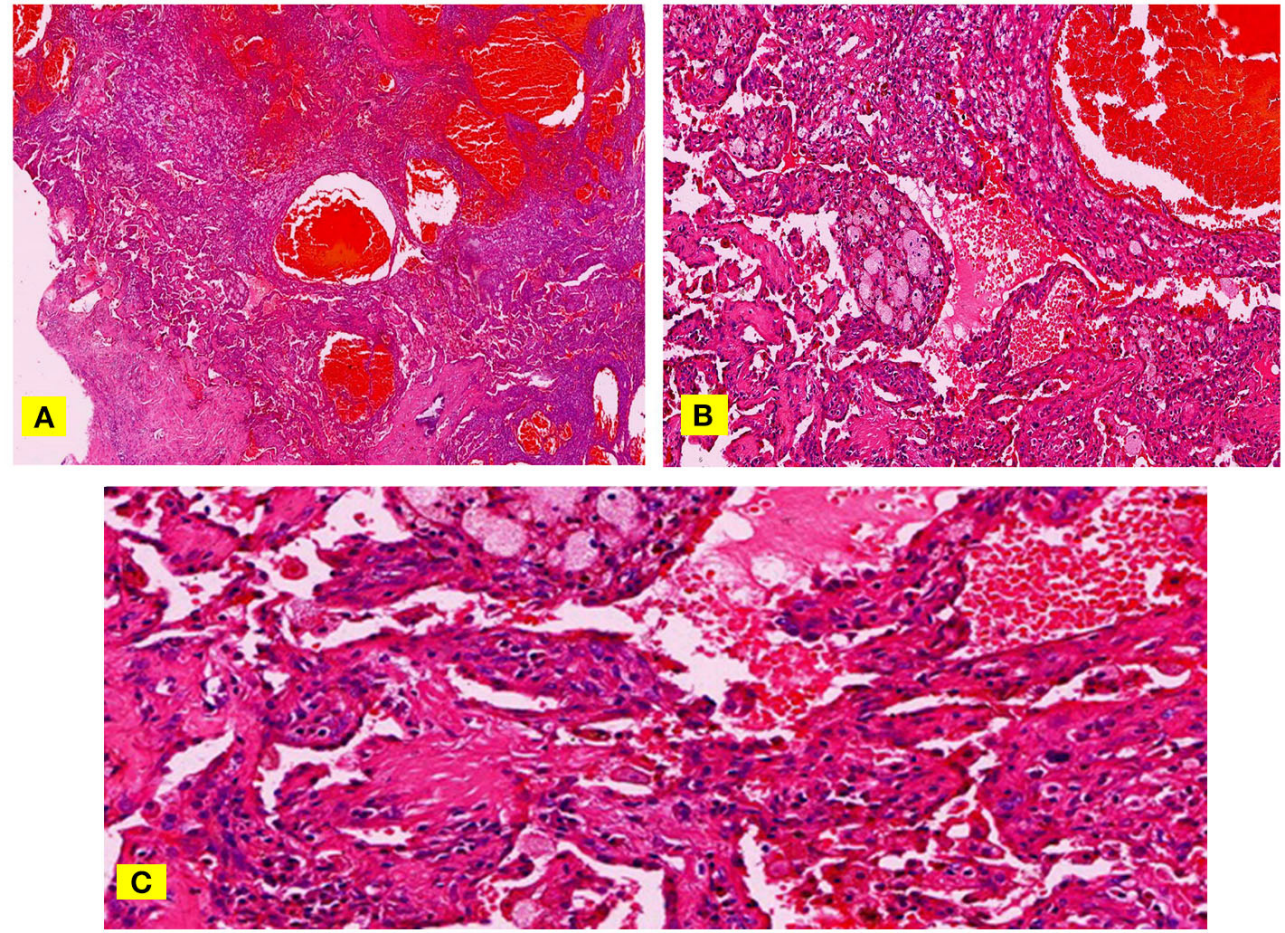
**(A–C)** H & E staining, papillary stromal and hemangioma-like area filled with a lot of fresh red blood cells **(A)**. At high magnification, some nipples are immature and migrate to the hemangioma-like areas **(B)**. The space between these immature nipples is more obvious and communicates with the hemangioma-like area **(C)**.

The shortest distance between the lesion margin and the pleura was between 0.00 and 29.16 mm. Among them, the lesion was grown attached to the pleura in 16 cases that was, the distance was 0.00 mm, the distance was 0–5 mm in 21 cases, 0–10 mm in 25 cases, 0–20 mm in 31 cases, and > 20 mm in three cases (Figure 3).

### The Relationship Between the Lesion Size and the Distance From the Pleura

Through the Logistic model analysis, there was no significant correlation between the lesion size and the shortest distance from the pleura (*R*^2^ = 0.066).

### MSCT Enhancement Manifestations

#### Enhancement Form, Degree, and Characteristics

**Figure 4:**
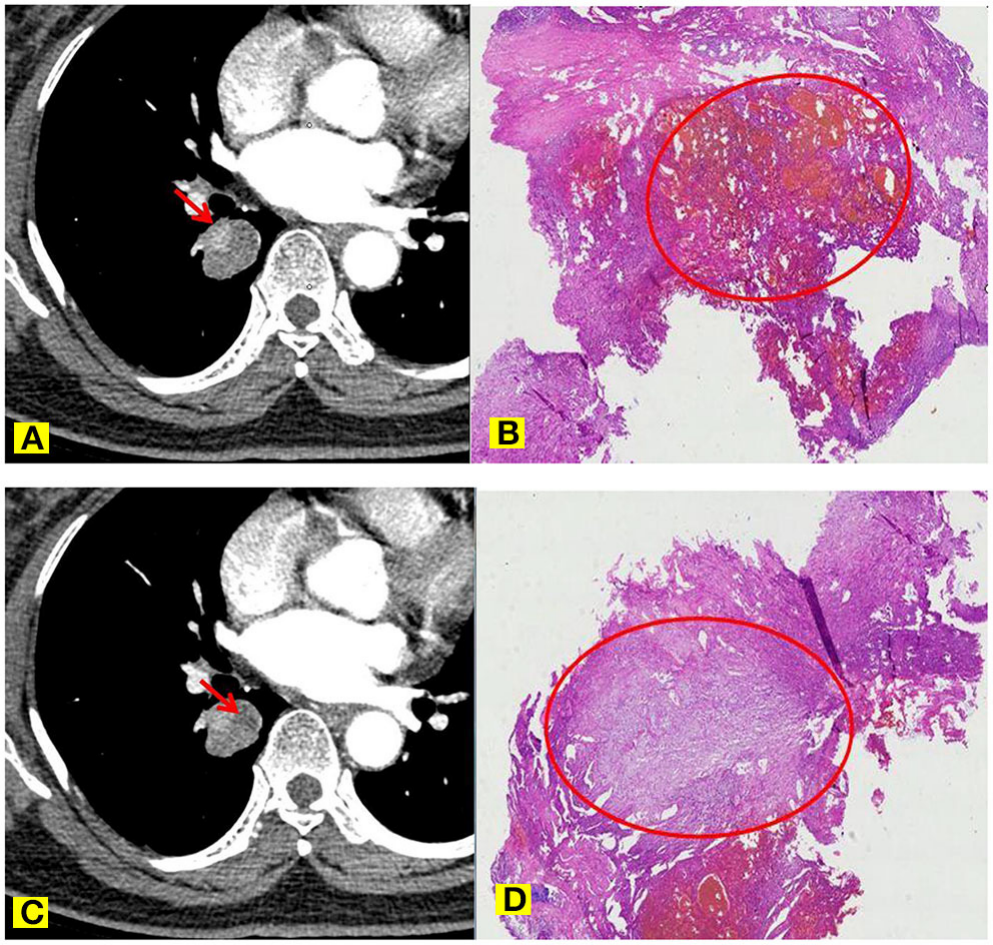
**(A–D)**. Comparison of MSCT imaging manifestations **(A)** and pathological features **(B)** of PSP nipple areas and hemangioma-like areas. Comparison of MSCT imaging manifestations **(C)** and pathological features **(D)** of PSP solid areas and sclerosing areas.

Among the 34 cases in this group, except for one PSP nodule with a long diameter of 8.58 mm, which was almost uniformly enhanced, the remaining cases showed uneven enhancement. The CT value of the apparent enhancement area in the lesion increased by an average of 67.06 HU, while the CT value of most lesions increased by 40–70 HU (Figure 4). The CT value of the slight enhancement area increased by an average of 16.22 HU, and the CT value of most lesions increased by 10–20 HU. The lesion was negatively correlated with the total degree of enhancement (*r* = −0.587, *p* < 0.01), and negatively correlated with the difference in volume between the obvious enhancement area and the slight enhancement area (*r* = −0.795, *p* < 0.01), that is, the smaller the lesion volume, the larger the obvious enhancement area, the smaller the slight enhancement area, the larger the lesion volume, the smaller the obvious enhancement area and the larger the slight enhancement area.

#### The Relationship Between Blood Vessels, Bronchus, and Lesions

Of the 34 cases in this group, 21 cases showed signs of welt vessel (61.7%), but none of these welt vessels entered the lesion, and there was no obvious correlation between the vascular-like enhancement area inside the lesion and the welt vessels outside the lesions. All the 24 cases had no bronchial entry into the lesions.

### Pathological Manifestations

#### Gross Specimen

The 34 cases of PSP in this group had clear boundaries and were easily dissociated from the surrounding lung tissues. The colors of the cut surface were apricot-white-gray to yellow-brown, with a medium texture. In some cases, dark red bleeding areas, hemosiderin deposition areas, and calcification have been observed. In all cases, no mediastinum, hilus pulmonis, parabronchial, or interlobar lymph node metastasis were observed.

#### Microstructures

One case showed that the sclerosing area was not obvious, and the remaining 33 cases showed all four growth patterns (nipple area, sclerosing area, solid area, and hemangioma-like area). None of the cases showed one growth pattern. Among 34 cases, the solid area was dominated in 11 cases (32.3%), the sclerosing area was dominated in 9 cases (26.4%), the hemangioma-like area was dominated in 10 cases (29.4%), and nipple area was dominated in four cases (11.7%).

### Immunohistochemistry

In all the 34 cases, immunohistochemical analysis showed positive expression of TTF-1 in polygonal cells and surface cubic cells.

## Evaluation of Imaging and Pathological Diagnosticians

The assessment results of the experimental group (nine points for imaging diagnosticians and eight points for pathological diagnosticians) were significantly higher than those for the control group (six points for imaging diagnosticians and five points for pathological diagnosticians). In particular, the accuracy of intraoperative frozen section diagnosis was 60% higher in the experimental group than that in the control group.

## Discussion

There were 33 females in this group of patients with PSP. Devouassoux-shishebonar et al. (27) investigated the immunohistochemistry of 100 cases of PSP and found that sex hormone receptors, especially progesterone receptors, were expressed in most of the PSP ring cells, suggesting that the high incidence in women was related to female hormones.

Statistics analysis revealed that all 34 cases of lesions in this group occurred in the lungs, with a peripheral type of 97%, in which 92% of the lesions (31/34) were within 20 mm from the pleura, and 46% of the lesions (16/34) were grown with attachment to the pleura (including mediastinal pleura and interlobular pleura). We believe that this sign can be used as one of the reliable features of preoperative imaging diagnosis for PSP. The histological reasons for this feature are currently unclear and may be related to the fact that PSP originates from type II alveolar epithelial cells, but further research is still needed.

Interestingly, lung adenocarcinoma, which also originates from type II alveolar epithelial cells, also occurs primarily in the extrapulmonary area. Of course, PSP and lung adenocarcinoma have obvious histological and imaging differences that will not be repeated here.

PSP originates from type II alveolar epithelial cells and is an intraparenchymal tumor with well clinical processes (2, 3). The growth pattern is the real expansive growth. It originates from a primary structure bud and can be continuously replicated based on that structure, pushing outwards. Since it is not invasive, theoretically, it is impossible to pass through natural pulmonary arteriovenous and bronchial tubes. Pathological data have confirmed that the hemangioma-like PSP components are not a real vascular structure, and there are no normal pulmonary blood vessels and bronchial structures in PSP. While reviewing the MSCT imaging findings of this group of patients, 15 cases showed welt vessel signs, but none of them entered the lesion. In all 24 cases, no bronchus came the lesion, and the results obtained from imaging analysis were fully consistent with the pathological features. Therefore, we believe that this sign can be used as the second reliable feature of PSP for preoperative imaging diagnosis.

Among the 34 cases in this group, except for one case of PSP nodule with a long diameter of 8.58 mm, that was almost uniformly enhanced, the remaining cases showed uneven enhancement. Besides, the smaller the volume of PSP lesions, the greater the proportion of obvious areas of enhancement, and the smaller the proportion of slight enhancement areas. In contrast, the larger the lesion volume, the smaller the proportion of obvious enhancement areas, and the higher the proportion of slight enhancement areas. Pathological specimens showed that the smaller the lesion volume, the higher the proportions of both hemangioma-like and nipple areas on the tissue configuration, and the lower the proportions of solid and sclerosing areas. In contrast, the larger the lesion, the higher the proportions of solid and the sclerosing regions, the lower the proportions of both hemangioma-like and nipple areas. By combining high magnification observations, fresh red blood cells with complete morphology can be seen in the interstitial space of the hemangioma-like regions and the nipple areas, suggesting that these gaps can be connected to the hemangioma-like areas through specific channels. In addition, the nipple areas are mostly migrating with the hemangioma-like areas, which can better explain the connectivity between the two. The essence of the hemangioma-like area is that a cavity is filled with a large volume of fresh blood, which includes many normal and intact red blood cells, indicating its fluidity feature. Moreover, there are tiny blood vessels in the interstitial space of the nipple areas, but the cross-section of the lumen is far less than the interstitial space. Combined with the MSCT imaging manifestation, the obvious contrast-enhanced area corresponded to the nipple and the hemangioma-like regions, and the slightly enhanced regions corresponded to the solid area and the sclerosing area.

This characteristic of PSP is due to the clear expression of CK, EMA, SP-B, and TTF-1 on the surface cubic cells, suggesting that they originate from alveolar type II cells or have a tendency to differentiate into alveolar type II cells (28). The polygonal cells in the interstitium have clear expressions of Vimentin, Syn, and weak EMA expression, indicating that the polygonal cells in the interstitium have the potential for multi-directional differentiation (29, 30). Nakatani et al. (31) and Wang Yan et al. (32) concluded through immunohistochemical staining of PSP that polygonal cells and surface cubic cells are tumor parenchymal cells in PSP tissues in different differentiation directions, and both have the characteristics of mutual migration in histology. As a result, PSP formation has undergone a process of evolution from hemangioma type to the nipple type, the solid type, and then to the sclerosing type. Combining the pathological specimens and MSCT imaging manifestations, the smaller lesions are mainly hemangioma-like and/or nipple types, so the enhancement is obvious. With the extension of the course of the disease and the increase of the lesion volume, the solid and sclerosing structures of the lesion gradually increase and are unevenly distributed, so that the degree of enhancement on MSCT in this area is lower, making the entire lesion appear unevenly enhanced. All 34 cases in this group have the abovementioned features. Therefore, we believe that this pathological feature of PSP and the derived MSCT imaging manifestations can be used as the third reliable feature of preoperative imaging diagnosis. There are several reports, which described other external and surrounding traditional MSCT manifestations of PSP tumors (such as welt vessel signs, halo sign, false capsule signs, air crescent signs, etc.), and this study did not repeat them here.

## Conclusion

As a common benign tumor of the lung parenchyma, PSP is frequently detected in middle-aged women. The right lung is more common than the left lung, while the inferior lobe is more common than the superior lobe. By studying the correlation between clinicopathological features and MSCT imaging manifestations, we believe that the following features are reliable imaging signs for the preoperative and intraoperative diagnosis of PSP. These imaging characteristics can be summarized as follows:

1. It appears as close to the pleura.
2. There is no correlation between the lesion and its surrounding blood vessels and bronchi.
3. After dynamic enhancement scanning, the lesion volume is negatively correlated with the range of obvious enhancement areas. With the extension of the disease and the increase of the lesion volume, the area of the obvious enhancement decreases, and the range of the slight enhancement area increases, making the whole lesion appear unevenly enhanced.
4. While analyzing pathological frozen sections, it is important to combine MSCT images, including enhanced scanning, round non-lobulated, and surrounding halo bleeding, which are found to be very intuitive features of the PSP.
5. There is no pulmonary artery and bronchus passing through in frozen sections.
6. The center of the PSP nipple structure is the polygonal cells rather than the fibrous blood vessel axis.

At the same time, the experimental group mastering the new diagnostic approach scored nine points (imaging diagnostician) and eight points (pathological diagnostician), respectively, while the control group using traditional knowledge scored six points (imaging diagnostician) and five points (pathological diagnostician). Therefore, while grasping the knowledge of traditional PSP imaging and pathological diagnosis, if we can further apply the new knowledge we have summarized to the clinic, the accuracy of preoperative and intraoperative diagnosis will be significantly improved. Of course, our assessment subjects are very limited, and there will be some errors in the statistical results, but it cannot cover up the fact that the new diagnostic method can significantly improve the accuracy of preoperative and intraoperative diagnosis of PSP.

While PSP is a benign tumor, there are few reports of case studies related to other malignant tumors or with hilar, mediastinum, peribronchial, and interlobular lymph node metastases. Dacic et al. (33) reported that the p53 gene on the 17q arm of the PSP chromosome showed a high frequency of loss of heterozygosity, which known to be a possible malignant tumor. Wang Yan (34) investigated 19 cases of PSP and found that the mutation rate of p53 genes in tissues was up to 15.8%, which was similar to that of other malignant tumors, suggesting that PSP may have possible malignant biological behaviors.

The malignant behaviors of PSP are expressed through lymph node metastasis (7), but these features have been reported to have no significant effect on the prognosis of patients (21, 35). Since the mechanisms of lymph node metastasis of PSP are still not clear, it is recommended that patients diagnosed with PSP and treated with surgery should be followed up for a long period of time. Although there is no reliable evidence of recurrence or metastasis in the remaining cases except four in which loss of follow-up occurred in this group, the outcome of this group still requires long-term follow-up.

## Data Availability Statement

The original contributions presented in the study are included in the article/supplementary material, further inquiries can be directed to the corresponding author/s.

## Ethics Statement

All experimental procedures of this study were approved by the local ethics committee of Xiangya Hospital, Central South University, Changsha, China (Code: 202006082) and were in accordance with the ethical standards and regulations of human studies of the Helsinki declaration (2014). The patients/participants provided their written informed consent to participate in this study.

## Author Contributions

GX and ZX conceived and designed research. GX, ZW, ZX, ML, WL, YX, and TM conducted experiments. ML and WL analyzed data. GX and ZX edited and wrote the final version of manuscript. All authors were involved in drafting the manuscript as well as revising it critically for relevant intellectual content. All authors read and approved the manuscript.

## Conflict of Interest

The authors declare that the research was conducted in the absence of any commercial or financial relationships that could be construed as a potential conflict of interest.
